# Supplementary material for: Vaccination with VLPs Presenting a Linear Neutralizing Domain of S. aureus Hla Elicits Protective Immunity
Source: Toxins (Basel). 2020 Jul 11;12(7):450. doi: 10.3390/toxins12070450 (PMC7404987; doi:10.3390/toxins12070450)
Supplement: Supplementary file 1 [file toxins-12-00450-s001.pdf]

Supplementary Materials: Vaccination with VLPs Presenting a Linear Neutralizing Domain of *S. aureus* Hla Elicits Protective Immunity

Jason A. Joyner, Seth M. Daly, Julianne Peabody, Kathleen D. Triplett, Srijana Pokhrel, Bradley O. Elmore, Diane Adebanjo, David S. Peabody, Bryce Chackerian and Pamela R. Hall

Supplemental Methods

Expression of LND in AP205 and LND conjugation efficiency to Qβ was confirmed by sodium dodecyl sulfate polyacrylamide gel electrophoresis (SDS PAGE) using a Bolt™ 4-12% Bis-Tris Plus gel (Invitrogen, Carlsbad, CA, USA) and stained with Coomassie Brilliant Blue R-250 (Bio-Rad, Hercules, CA, USA). Assembly of VLP, indicated by RNA encapsidation and protein colocalization, was confirmed by agarose gel electrophoresis (1%) followed by ethidium bromide and coomassie staining, respectively. Purified VLP were passed through a 0.2 μm filter to ensure sterility for mouse vaccinations. VLP concentration was determined by comparison against lysozyme standards on SDS-PAGE analyzed with Image Studio™ Lite (LI-Cor Biosciences, Lincoln, NE, USA).

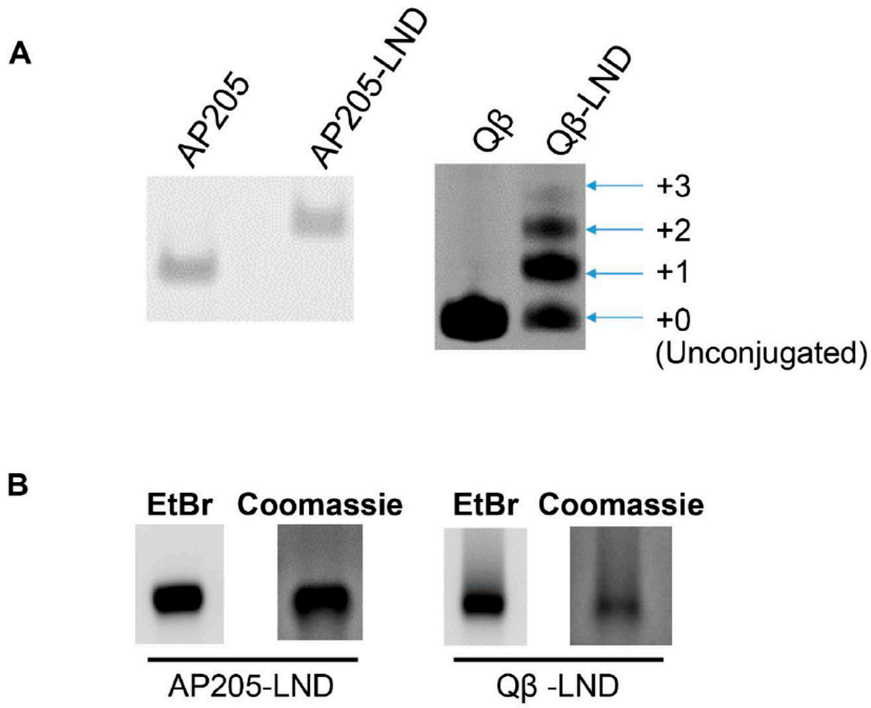

Figure S1. Validation of AP205-LND and Qβ-LND VLPs. (A) SDS-PAGE showing protein bands for monomers of (left) AP205 and AP205 with LND genetically incorporated, or (right) Qβ and Qβ chemically conjugated to Hla LND peptide. **(B)** Agarose gels stained with ethidium bromide, showing encapsidation of RNA or with coomassie showing VLP coat protein colocalization with encapsidated RNA for intact AP205-LND VLPs and Qβ-LND VLPs (left and right, respectively).
